# Reporting quality of pilot clinical trials in chronic kidney disease patients on hemodialysis: a methodological survey

**DOI:** 10.1186/s40814-019-0436-3

**Published:** 2019-04-06

**Authors:** Sarah Daisy Kosa, Jillian Monize, Alvin Leenus, Selvin Leenus, Simranjit Samra, Sylwia Szwiega, Daniel Shi, Sara Valvasori, Amiram Gafni, Charmaine E. Lok, Lehana Thabane

**Affiliations:** 1 Kidney CARE Network International, Toronto, Ontario Canada; 2 Division of Nephrology, Toronto General Hospital, University Health Network, 200 Elizabeth Street 8N844, Toronto, Ontario M5G 2C4 Canada; 3 Department of Health Research, Evidence, and Impact, McMaster University, Hamilton, Ontario Canada; 4 Faculty of Health Sciences, McMaster University, Hamilton, Ontario Canada; 5 Faculty of Health Sciences, Queen’s University, Kingston, Ontario Canada; 6 Biostatistics Unit, St Joseph’s Healthcare, Hamilton, Ontario Canada

**Keywords:** Pilot trials, Feasibility trials, Reporting quality, Transparency, Guideline adherence, Hemodialysis, CONSORT

## Abstract

**Background:**

The conduct of high-quality pilot studies can help inform the success of larger clinical trials. Guidelines have been recently developed for the reporting of pilot trials.

**Objective:**

This methodological survey evaluates the completeness of reporting in pilot randomized controlled trials in chronic kidney disease patients on hemodialysis (HD patients) and explores factors associated with better completion of reporting.

**Methods:**

The authors searched Pubmed on July 1, 2018, for all pilot trials conducted in HD patients. Reporting quality was assessed against the 40-item Consolidated Standards of Reporting Trials (CONSORT) Extension for Pilot Trials. Study factors including year and country of publication, intervention, number of centers, type of funding, and journal endorsement of CONSORT were also examined.

**Results:**

The mean number of items reported from the CONSORT extension for pilot trials across all included articles was 18.4 (standard deviation [SD] = 4.4). In the adjusted analysis, studies reported in later years (IRR = 1.026, 95% CI [1.018, 1.034], *p* < 0.001) and an increase of 20 persons in sample size (adjusted IRR = 1.021, 95% CI [1.010, 1.031], *p* < 0.001) were associated with a significantly higher number of CONSORT pilot items reported.

**Conclusions:**

Current reporting completeness of pilot trials in HD patients is suboptimal. Endorsing the CONSORT extension specific to pilot and feasibility studies and ensuring that pilot trials focus on the feasibility objectives may improve reporting completeness of these trials.

**Electronic supplementary material:**

The online version of this article (10.1186/s40814-019-0436-3) contains supplementary material, which is available to authorized users.

## Background

Chronic kidney disease (CKD) is a significant and growing global health problem, with a prevalence estimated to be between 11 to 13% [1]. CKD is defined as having a decreased kidney function for at least 3 months, regardless of the etiology, and has many serious complications such as uremia, volume overload, and hematologic and metabolic disturbances [2, 3]. Risk of mortality, largely due to cardiovascular disease, is significantly increased in the CKD population and increases as kidney function declines [2]. An estimated two million CKD patients with very little or no residual kidney function require kidney replacement therapy [4]. Kidney replacement therapy is very costly, consuming 6.7% of the total Medicare budget to care for less than 1% of the covered population. The costliest modality is hemodialysis (HD), estimated at over US$87,000 per year per patient [5]. Despite significant investment into research in the last 40 years, survival and quality of life of patients on HD remain low [6].

There are many pressing clinical questions in HD which require a definitive, well-powered randomized clinical trial (RCT) [7]. Health care providers and patients alike have identified the need for further research across a range of priorities in HD including how to best address vascular access problems, reduce fatigue, risk of mortality, and cardiovascular disease and to improve dialysis adequacy [6]. However, the immense quantity of information and resources required for the conduct of adequately powered RCTs across these clinical areas in HD can act as a barrier to their conduct [7, 8]. Pilot studies can facilitate designing such definitive RCTs by assessing feasibility of screening, recruitment, coordination and acceptability, safety, and fidelity of the intervention and the study protocol, as well as informing power calculations [7, 9, 10].

However, pilot trial methodology is often criticized for inadequacies, mainly critiquing the emphasis on hypothesis testing and the lack of criteria for evaluating feasibility [11, 12]. To address these issues, the Consolidated Standards of Reporting Trials (CONSORT) extension for reporting randomized pilot and feasibility trials was published in 2016, which builds on the statement published in 2010 [13]. The 2016 CONSORT extension, which will be utilized in this study, lays the groundwork for the reporting of pilot trials, as well as informs their design and implementation. This is expected to enhance the completeness and transparency in the reporting of pilot RCTs and establish a standardized approach to this area of research [13].

Until recently, standards for the reporting of pilot studies have been unavailable and it is likely that there are significant gaps and inconsistencies in the reporting of pilot studies in HD—as similarly identified in other areas of clinical research [14]. Identification of these gaps may not only help inform initiatives to improve reporting, but also potentially raise awareness among clinicians and research about pilot trial design and implementation. Using the newly published CONSORT extension for pilot trials, we undertook a methodological survey to assess reporting completeness among pilot trials investigating interventions in CKD patients on hemodialysis (HD patients) and explored factors associated with better completion of reporting. A methodologic review is a type of study designed to examine methodologic quality of a sample of articles, generally within a certain discipline or study design [14–16].

## Methods

### Study eligibility

Inclusion criteria: (1) the words “pilot” or “feasibility” used to describe its design, (2) randomized control trial, (3) examine interventions in HD patients, and (4) published in English.

Exclusion criteria: (1) single-arm observational pilot or feasibility studies, (2) quasi-randomized trials, and (3) studies among nonclinical or acute populations.

### Search strategy

In order to survey the medical literature for pilot trials in HD patients, we searched Medline/Pubmed and included studies published before July 1, 2018, using the following strategy: (1) feasibility studies/ or pilot projects/; (2) pilot stud*.mp.; (3) exp Randomized Controlled Trial/; (4) randomized controlled trial.pt.; (5) controlled clinical trial.pt.; (6) randomized controlled trials.sh.; (7) random allocation.sh.; (8) double-blind method.sh.; (9) single-blind method.sh.; (10) 3 or 4 or 5 or 6 or 7 or 8 or 9; (11) (animals not humans).sh.; (12) 10 not 11; (13) 1 or 2; (14) 12 and 13; (15) exp Renal Dialysis/; (16) renal dialysis.tw.; (17) 15 or 16; (18) hemodialysis.tw.; (19) haemodialysis.tw.; (20) 17 or 18 or 19; (21) exp peritoneal dialysis/; and (22) 20 not 21.

### Study selection

One author (SK) screened all titles and abstracts based on the inclusion and exclusion criteria and conducted a full-text review of randomly selected citations to assess eligibility. The full-text articles were then screened independently and in duplicate for eligibility by three teams of reviewers (AL and SL, DS and SV, and SS and SS).

### Outcome measures

The primary outcome of this survey was the completeness of reporting of each of the items on CONSORT statement extension for randomized pilot and feasibility trials checklist, measured as a number and proportion of studies reporting each of the 40 items. The secondary outcome was the completeness of reporting of the CONSORT statement extension for randomized pilot and feasibility trials checklist, as measured by the total number of applicable items reported.

### Data extraction

The Excel-based data extraction form was developed based on a previous methodological survey [16] and collected study characteristics including year and country of publication, sample size, number of sites, type of funding (i.e., industry, non-industry), type of intervention (i.e., pharmaceutical, behavioral/educational, dialysis technology/technique, nutritional supplements, vascular access technology/technique, and other non-pharmaceutical interventions), whether the manuscript explicitly stated the pilot study to be prelude to definitive study (i.e., yes, no), journal endorsement of CONSORT statement if the article was published after 2010 (i.e., yes, no), and reporting of individual items on the CONSORT extension for pilot and feasibility studies (Additional file 1). For the reporting of individual items on the CONSORT extension for pilot and feasibility studies, each item on the checklist was scored as either “reported” or “not reported,” indicating whether or not the article reported the appropriate information as per the criteria outlined in the original publication (Table 1). Exceptions to this are items 6c, 7b, 11a, 11b, 18, and 19a, all of which had “not applicable” as an option [13].

**Table 1:** Reporting of items on the CONSORT Extension for Pilot and Feasibility Trials

CONSORT Items	*n*	%	Lower 95% CI	Upper 95% CI	*N*
Title and Abstract					
1a. Identification as a pilot or feasibility randomized trial in the title	52	60.5%	49.9%	70.3%	86
1b. Structured summary of pilot trial design, methods, results, and conclusions (for specific guidance see CONSORT abstract extension for pilot trials)	79	91.9%	84.7%	96.3%	86
Introduction					
2a. Scientific background and explanation of rationale for future definitive trial, and reasons for randomized pilot trial	23	26.7%	18.3%	36.8%	86
2b. Specific objectives or research questions for pilot trial	84	*97.7%*	92.7%	99.5%	86
Methods					
Trial design					
3a. Description of pilot trial design (such as parallel, factorial) including allocation ratio	36	41.9%	31.8%	52.4%	86
3b. Important changes to methods after pilot trial commencement (such as eligibility criteria), with reasons	5	**5.8%**	2.3%	12.3%	86
Participants					
4a. Eligibility criteria for participants	80	*93.0%*	86.2%	97.0%	86
4b. Settings and locations where the data were collected	57	66.3%	55.9%	75.6%	86
4c. How participants were identified and consented	58	67.4%	57.1%	76.6%	86
Interventions					
5. The interventions for each group with sufficient details to allow replication, including how and when they were actually administered	74	86.0%	77.6%	92.1%	86
Outcome measurement					
6a. Completely defined prespecified assessments or measurements to address each pilot trial objective specified in 2b, including how and when they were assessed	79	91.9%	84.7%	96.3%	86
6b. Any changes to pilot trial assessments or measurements after the pilot trial commenced, with reasons	3	**3.5%**	1.0%	9.0%	86
6c. If applicable, prespecified criteria used to judge whether, or how, to proceed with future definitive trial*	7	9.0%	4.1%	16.8%	78
Sample size					
7a. Rationale for numbers in the pilot trial	24	27.9%	19.3%	38.0%	86
7b. When applicable, explanation of any interim analyses and stopping guidelines*	3	**7.9%**	2.3%	19.6%	38
Randomization					
8a. Method used to generate the random allocation sequence	30	34.9%	25.4%	45.3%	86
8b. Type of randomization(s); details of any restriction (such as blocking and block size)	24	27.90%	19.3%	38.0%	86
Allocation concealment mechanism					
9. Mechanism used to implement the random allocation sequence (such as sequentially numbered containers), describing any steps taken to conceal the sequence until interventions were assigned	22	25.60%	17.3%	35.5%	86
Implementation					
10. Who generated the random allocation sequence, who enrolled participants, and who assigned participants to interventions	16	18.60%	11.5%	27.8%	86
Blinding					
11a. If done, who was blinded after assignment to interventions (for example, participants, care providers, those assessing outcomes) and how*	33	43.40%	32.7%	54.6%	76
11b. If relevant, description of the similarity of interventions*	20	71.40%	53.2%	85.5%	28
Statistical methods					
12. Methods used to address each pilot trial objective whether qualitative or quantitative	84	*97.70%*	92.7%	99.5%	86
Results					
Participant flow					
13a. For each group, the numbers of participants who were approached and/or assessed for eligibility, randomly assigned, received intended treatment, and were assessed for each objective	62	72.10%	62.0%	80.7%	86
13b. For each group, losses and exclusions after randomization, together with reasons	66	76.70%	67.0%	84.7%	86
Recruitment					
14a. Dates defining the periods of recruitment and follow-up	37	43.00%	32.9%	53.6%	86
14b. Why the pilot trial ended or was stopped	8	9.30%	4.5%	16.8%	86
Baseline data					
15. A table showing baseline demographic and clinical characteristics for each group	72	83.70%	74.9%	90.4%	86
Numbers analyzed					
16. For each objective, number of participants (denominator) included in each analysis. If relevant, these numbers should be by randomized group	39	45.30%	35.1%	55.9%	86
Outcomes and estimation					
17. For each objective, results including expressions of uncertainty (such as 95% confidence interval) for any estimates. If relevant, these results should be by randomized group	70	81.40%	72.2%	88.5%	86
Ancillary analyses					
18. Results of any other analyses performed that could be used to inform the future definitive trial*	13	21.30%	12.5%	32.8%	61
Harms					
19. All important harms or unintended effects in each group (for specific guidance see CONSORT for harms)	48	55.80%	45.3%	66.0%	86
19a. If relevant, other important unintended consequences*	19	27.90%	18.4%	39.4%	68
Discussion					
Limitations					
20. Pilot trial limitations, addressing sources of potential bias and remaining uncertainty about feasibility	63	73.30%	63.2%	81.7%	86
Generalisability					
21. Generalisability (applicability) of pilot trial methods and findings to future definitive trial and other studies	38	44.20%	34.0%	54.7%	86
Interpretation					
22. Interpretation consistent with pilot trial objectives and findings, balancing potential benefits and harms, and considering other relevant evidence	84	*97.70%*	92.7%	99.5%	86
22a. Implications for progression from pilot to future definitive trial, including any proposed amendments	14	16.30%	9.6%	25.1%	86
Other information: Registration					
23. Registration number for pilot trial and name of trial registry	27	31.40%	22.3%	41.7%	86
Protocol					
24. Where the pilot trial protocol can be accessed, if available	7	**8.10%**	3.7%	15.3%	86
Funding					
25. Sources of funding and other support (such as supply of drugs), role of funders	61	70.90%	60.8%	79.7%	86
26. Ethical approval or approval by research review committee, confirmed with reference number	53	61.60%	51.1%	71.4%	86

Note: Italics indicates bottom 10%, and bold indicates top 10%

*Studies for which the item was not applicable were not included in the total *N*

*N* = total number of studies (i.e., numerator); *n* = count

### Data analysis

All statistical analyses were performed in SPSS version 25.

#### Descriptive statistics

The completeness of reporting was summarized using descriptive statistics percentages for the general characteristics and number of articles reporting each CONSORT statement item (for items 6c, 7b, 11a, 11b, 18, and 19a, the percentage was calculated based on the total number of studies for which the item was applicable). The mean, standard deviation, and range for the total number of CONSORT statement items reported, sample size, and number of sites were also calculated. To calculate the mean number of CONSORT items reported, “not applicable” responses to reporting items 6c, 7b, 11a, 11b, 18, and 19a were excluded.

#### Inferential statistics

We conducted a Poisson regression to explore factors, including year of publication, sample size, multisite study (yes or no), industry funding (yes or no), prelude to a definite trial (yes or no), and journal endorsement of CONSORT (yes or no), associated with completeness of reporting as measured by the number of reported CONSORT items (“not applicable” items were excluded). Based on previous research, we hypothesized that a later publication date [17], larger sample size [18], multisite study [16], industry funding [16, 18, 19], and journal endorsement of CONSORT [20] would be associated with better reporting. The results of the Poisson regression were reported as unadjusted and adjusted incidence rate ratios (IRR) including 95% confidence interval (CI) and *p* value (*α* = 0.05).

Journal of publication was also considered as a potential factor to include in the Poisson regression as some clustering has been observed in prior studies on reporting [16, 18]; however, no clustering within journals was noted in exploratory analyses, likely due to the wide breadth of journals in which the included studies were published, with no more than 10 studies published in the same journals (see characteristics of included studies below).

## Results

**Fig. 1:**
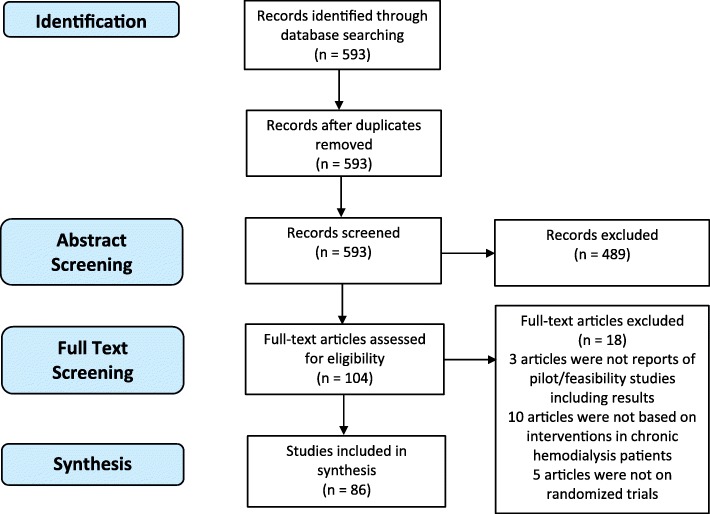
PRISMA 2009 Flow Diagram

Our initial search retrieved 593 records, of which 86 were included in the synthesis (see Fig. 1 for PRISMA diagram).

### Characteristics of included studies

The studies examined a wide range of interventions (see Table 2) including pharmaceutical (33.7%, e.g., magnesium carbonate plus calcium acetate, oral cholecalciferol), behavioral/educational (19.8%, e.g., cycling, resistance, or cycling and resistance, chairside meditation), dialysis technology/technique (17.4%, e.g., equilibrated kt/v goal of 1.4, fx-e membrane), nutritional supplements (14.0%, e.g., fish oil), vascular access technology/technique (12.8%, e.g., dialysis needles placed 2.5 cm and then 5 cm apart, u clip anastomosis), and other non-pharmaceutical interventions (2.3%, i.e., acupuncture, low-intensity vibration device). The majority of the studies were single center (68.6%) and had a sample size of 50 or less (74.4%). The mean sample size was 44.0 (standard deviation (SD) = 55.1, range = 4–448) and number of sites was 2.2 (SD = 2.7, range = 1–15).

**Table 2:** Characteristics of included articles

Characteristics		Count	%
Intervention type (*N* = 86)	Behavioral/educational	17	19.8%
	Dialysis technology/technique	15	17.4%
	Non-pharmaceutical other	2	2.3%
	Nutritional supplement	12	14.0%
	Pharmaceutical	29	33.7%
	Vascular access technology/technique	11	12.8%
Number of sites (*N* = 86)	Multi center	27	31.4%
	Single center	59	68.6%
Sample size (*N* = 86)	Greater than 50	22	25.6%
	50 or less	64	74.4%
Industry funded (*N* = 86)	Yes	26	30.2%
	No	60	69.8%
Location of study by continent (*N* = 86)	Africa	1	1.2%
	Asia	13	15.1%
	Australia/New Zealand	6	7.0%
	Europe	24	27.9%
	North America	42	48.8%
Year of publication (*N* = 86)	1990–1995	1	1.2%
	1996–2000	8	9.3%
	2001–2005	10	11.6%
	2006–2010	15	17.4%
	2011–2015	38	44.2%
	2016–2018	14	16.3%
Journal endorses CONSORT (only published in 2010 or later)* (*N* = 56)	Yes	24	42.9%
	No	32	57.1%
Journal endorses CONSORT (all years, all published before 2010 set to No)** (*N* = 86)	Yes	24	27.9%
	No	62	72.1%
Prelude to definitive trial (*N* = 86)	Yes	15	17.4%
	No	71	82.6%

*CONSORT Consolidated Standards of Reporting Trials; only includes studies published in 2010 or later

**Includes all studies, with all studies published before 2010 set to No

The studies were conducted across the globe, including Africa (1.2%), Asia (15.1%), Australia/New Zealand (7.0%), Europe (27.9%), and North America (48.8%). The majority of studies were published since 2010 (60.5%). Among those published since 2010, 42.9% were published in journals that endorse the CONSORT statement. Only 17.4% of studies explicitly indicated in the manuscript that the study was a prelude to a definitive trial. The studies were published across a wide range of journals, with 10 studies published in *J Ren Nutr*, 8 in *Am J Kidney Dis*, 5 in *Nephrol Dial Transplant*, 4 in *J Vasc Access*, 3 in each of *Clin J Am Soc Nephrol*, *J Nephrol*, *Nephrology* (*Carlton*), and *Ther Apher Dial*, 2 in each of *Int Urol Nephrol*, *Kidney Int*, *Pharmacotherapy*, and *PLoS One*, with the remaining 39 studies published in different journals.

### Evaluation of reporting quality based on CONSORT extension

The mean CONSORT reporting score across all included articles was 18.4 (SD = 4.4, minimum = 8, maximum = 29) out of a possible 34 items (6c, 7b, 11a, 11b, 18, and 19a were excluded as they had a not applicable option). Table 1 shows the level of reporting of individual CONSORT items. The items reported by the largest proportion of articles (top 10%) were “2b. Specific objectives or research questions for pilot trial” (97.7%); “4a. Eligibility criteria for participants” (93.0%); “12. Methods used to address each pilot trial objective whether qualitative or quantitative” (97.7%); and “22. Interpretation consistent with pilot trial objectives and findings, balancing potential benefits and harms, and considering other relevant evidence” (97.7%).

The most poorly reported items (bottom 10%) were “3b. Important changes to methods after pilot trial commencement (such as eligibility criteria), with reasons” (5.8%); “6b. Any changes to pilot trial assessments or measurements after the pilot trial commenced, with reasons” (3.5%); “6c. When applicable, explanation of any interim analyses and stopping guidelines” (7.9%); and “24. Where the pilot trial protocol can be accessed, if available” (8.1%).

### Factors related to reporting of CONSORT extension items

Table 3 shows the unadjusted and adjusted IRRs for overall CONSORT reporting by study characteristics. In comparing the total number of reported CONSORT items by the prespecified study characteristics, studies reported in later years (adjusted IRR = 1.026, 95% CI [1.018, 1.034], *p* < 0.001) and an increase of 20 persons in sample size (adjusted IRR = 1.021, 95% CI [1.010, 1.031], *p* < 0.001) had a significantly higher number of CONSORT pilot items reported. After adjusting for other covariates and factors, the remaining study characteristics were not significantly associated with reporting completeness.

**Table 3:** Incidence rate ratios for the total number of CONSORT Pilot Trial Extension Items Reported

Variable	Unadjusted incident rate ratio (95% confidence interval)	*P* value	Adjusted incident rate ratio (95% confidence interval)	*P* value
Year	1.025 (1.017, 1.032)	< 0.001	1.026 (1.018, 1.034)	< 0.001
Sample size (1 unit increase = 20 participants)	1.019 (1.004, 1.035)	0.011	1.021 (1.010, 1.031)	< 0.001
Multisite study (single center)	0.966 (0.870, 1.073)	0.520	1.030 (0.941, 1.128)	0.517
Industry funding (non-industry funding)	1.026 (0.922, 1.142)	0.639	0.976 (0.880, 1.082)	0.639
Prelude to future definitive trial (not a prelude)	1.070 (0.909, 1.260)	0.416	1.033 (0.900, 1.185)	0.645
Journal endorses CONSORT (does not endorse)	1.122 (1.001, 1.258)	0.049	0.978 (0.870, 1.099)	0.704

## Discussion

In this systematic survey, one of the few to examine the completeness of reporting in pilot and feasibility RCTs [14], we found that the mean number of CONSORT extension items for pilot and feasibility studies was 18.4 out of a possible 34 applicable items. We did find, however, that there is a 2.6% increase in the number of CONSORT pilot items reported for each additional year of publication. Larger sample sizes were also associated with higher number of CONSORT pilot items reported; however, the observed effect was relatively small (e.g., there is a 2.1% increase in reporting completeness for an increase in sample size of 20 participants). The number of reported items may continue to improve over time, particularly with the 2016 publication of the CONSORT extension for pilot and feasibility RCTs. Indeed, the completeness of reporting full RCTs in nephrology has improved over time, though reporting quality issues for certain CONSORT (2010) items such as clinical trial design, mode of randomization, and intention-to-treat analysis persist [8, 21–23]. Lack of available guidelines prior to the CONSORT extension and lack of its awareness (only recently published) could be contributing to this suboptimal reporting [13]. These guidelines may help clarify the purpose and reporting of pilot studies in HD patients.

In our sample of pilot studies, we found that the reporting of some items was consistently reported. These items are very similar to those included in the 2010 CONSORT statement, and include specific objectives for the pilot trial, eligibility criteria for participants, methods used to address each pilot trial objective whether qualitative or quantitative, and the interpretation is consistent with pilot trial objectives and findings. However, certain items specific to pilot trials and clinical trial conduct transparency were reported in less than 10% of included studies which, as previously indicated, may be attributable to lack of reporting guidelines for pilot trials until recently. These include changes to methods and assessments or measurements after pilot trial commencement, progression criteria for a future definitive trial, and where the pilot trial protocol can be accessed. These items are critical to the development of a larger, definitive trial that is rigorously designed and well powered, which should be the primary purpose of a pilot study [9]. The low levels of reporting for these items is consistent with our finding that only 17.4% of included studies indicated that they were a prelude to a larger trial, as well as the findings of a previous study on reporting completeness in pilot trials in behavioral interventions (13%) [14]. The manuscripts may not have explicitly stated the pilot study to be a prelude to a definitive study (CONSORT 2a and 22a) as this might be assumed by authors given that it is the purpose of a pilot study; however, this should be explicitly reported for every study as recommended by the guidelines.

Many of the included studies were primarily designed to address questions of clinical efficacy of a wide range of interventions. Given the relatively small sample of the majority of the included trials, these studies were likely underpowered to do so. Though some items were well reported, going forward, it is critical that journals publishing pilot trials in HD patients ensure that these studies adhere to the pilot trial extension to the CONSORT reporting statement, as well as confirm that the primary objectives of these studies are related to feasibility—not efficacy—objectives. This may be accomplished as part of the peer review process or as a requirement of submission.

There are several limitations of this study that are important to acknowledge. We only included English-language studies due to feasibility purposes, which may limit the generalizability of these findings. As this was a methodological survey and not a systematic review, this study’s search was restricted to PubMed. Additionally, there may have been factors important to consider in completeness of reporting that we failed to capture in this study.

## Conclusions

Pilot and feasibility trials of interventions in HD patients can help to inform the design of well-powered clinical trials to address critical challenges in HD today and prevent waste of resources on poorly designed trials [22, 24]. The pilot studies conducted in this patient population over the last two decades examine a wide range of interventions from pharmaceutical to behavioral; however, they were largely not preludes to larger trials. Improving the reporting completeness of these trials through promotion and endorsement of the CONSORT extension specific to pilot and feasibility trials and ensuring that pilot trials focus on feasibility objectives may improve the utility of these pilot trials. Such pilot studies are needed in HD patients to help inform and design interventional trials that are “…sufficiently robust to provide reliable answers and are not constrained by inappropriate complexities in design or conduct” ([24], p., 297).

## Additional file

Additional file 1: Pilot clinical trials in chronic kidney disease patients on hemodialysis. The sample of pilot clinical trials in chronic kidney disease patients on hemodialysis included in the methodological survey. The data dictionary is included in the second tab. (XLS 158 kb)
